# Tuberculosis in childhood: a systematic review of national and international guidelines

**DOI:** 10.1186/1471-2334-14-S1-S3

**Published:** 2014-01-08

**Authors:** Elettra Berti, Luisa Galli, Elisabetta Venturini, Maurizio de Martini, Elena Chiappini

**Affiliations:** 1 Department of Health Sciences, Meyer Children University Hospital, University of Florence, Florence, Italy

## Abstract

**Background:**

Paediatric tuberculosis (TB) represents a major public health concern worldwide. About 1 million children aged less than 15 years develop TB each year, contributing to 3-25% of the total TB caseload. The aim of this review is to evaluate national and international guidelines concerning tuberculosis in childhood and compare them in terms of diagnosis and treatment strategies.

**Methods:**

A literature search of the Pubmed database was performed from January 2000 to August 2013, using the terms “tuberculosis” and “children”. The search was limited to guidelines and consensus conferences, human species and full text availability, with no language restrictions.

**Results:**

Twenty-seven national and international guidelines are identified. Several discrepancies on the diagnosis workup of TB are underlined. The main points of disagreement are represented by the interpretation of tuberculin skin test (TST) result and the recommendations on the use of TST and/or interferon-gamma release assay (IGRA) for the diagnosis of TB infection. Otherwise, all guidelines are in agreement that a microbiological confirmation should always be sought. Similarly, susceptibility drug testing and genotyping should be performed whenever it is possible on the basis of resources availability. On the contrary, the use of nucleic acid amplification tests (NAATs) for the *M. tuberculosis* detection is still controversial. A general consensus exists, otherwise, on TB treatment and only minor discrepancies are evidenced, such as the recommendations on daily or intermittent treatment regimens.

**Conclusions:**

Despite advances in TB diagnostic tools have been reached during the last decade, a lack of uniformity in their availability, indication and interpretation has relevant consequences for clinical practice. Further studies need to be performed to clarify this issue and identify a reliable and reproducible diagnostic workup. Moreover, future studies should analyze the drug metabolism and the efficacy of intermittent dosing regimes in childhood, as well as new treatment regimens in order to improve the therapy compliance.

## Background

Paediatric tuberculosis (TB) represents a major public health concern worldwide. The World Health Organization (WHO) reported that about 9 million people develop TB each year, and of whom about 1 million (11%) occur in children aged less than 15 years. Children contribute to 3–6% of the total TB caseload in developed countries and to more than 25% of the burden of TB disease in developing countries [1]. Nonetheless, paediatric TB has been relatively neglected for several years, mainly due to difficulties in mycobacterium isolation in children. Novel developments in diagnosis and treatment of paediatric TB have been carried out and, more recently, the definition of an adequate management of TB in childhood has become one of the main aspects of the global TB control efforts [1-4].

The aim of the present systematic review is to evaluate and compare national and international guidelines concerning diagnosis and treatment strategies in paediatric TB.

## Methods

A literature search of the Pubmed database was performed from January 2000 to August 2013, using the terms “tuberculosis” and “children” as key words. The search was limited to type of article, guideline and consensus conference, human species and full text availability, with no limits of language. The results were also restricted on the basis of title and abstract. Therefore, 23 articles were identified and of those 17 were considered relevant to the goal of the research.

In order to verify the completeness of the Pubmed database, a literature search of other databases (Web of Science, Embase, Pascal and National Guideline Clearinghouse) was also conducted, but the results were virtually overlapping.

Additionally, the websites of relevant government organizations and professional societies were reviewed for documents of interest (WHO, Center for Disease Control and Prevention - CDC, European Center for Disease Control and Prevention - ECDC, National Institute for Health and Care Excellence - NICE, American Academy of Pediatrics - AAP, International Union Against Tuberculosis and Lung Disease - IUATLD, American Thoracic Society - ATS, Canadian Thoracic Society - CTS). All reference lists were examined in order to identify pertinent publications, which were added in the review.

All the results were closely evaluated and the papers that were not pertinent or redundant were excluded. In particular, our search was limited to guidelines developed at national or international levels, excluding local or regional recommendations, and overall 27 articles were included in this review.

## Results

The twenty-seven guidelines identified through the search strategy are listed in table 1. Twelve guidelines regard exclusively paediatric TB [1,5,9-12,23-26,28-30], whereas 15 guidelines analyze the management of TB in both adults and children [6-8,13-22,27,31]. The majority of the guidelines were focused on restricted fields concerning paediatric TB, as diagnostic tools or treatment strategies, whereas eight guidelines discuss extensively all aspects related to the management of TB in childhood [1,9,12,18,22,28,29,31].

**Table 1:** National and international guidelines concerning paediatric tuberculosis.

Guideline Title [Reference]	Guideline Developer(s)	Countries or Regions	Year	Target Population	Guideline Fields
Guidance for national tuberculosis programmes on the management of tuberculosis in children [1]	WHO	International	2006	Children	Diagnosis Treatment

Rapid advice. Treatment of tuberculosis in children [5]	WHO	International	2010	Children	Treatment

Use of tuberculosis interferon-gamma release assays (IGRAs) in low- and middle-income countries: policy statement [6]	WHO	International	2011	Adults Children	Diagnosis

Policy statement: automated real-time nucleic acid amplification technology for rapid and simultaneous detection of tuberculosis and rifampicin resistance: Xpert MTB/RIF system [7]	WHO	International	2011	Adults Children	Diagnosis

Guidelines for the programmatic management of drug-resistant tuberculosis [8]	WHO	International	2011	Adults Children	Treatment

Desk-guide for diagnosis and management of TB in children [9]	IUATLD	International	2010	Children	Diagnosis Treatment

Evaluation of Tuberculosis Diagnostics in Children [10,11]	Expert Panel	International	2012	Children	Diagnosis

Tuberculosis (in: Red Book 2012) [12]	AAP	U.S.	2012	Children	Diagnosis Treatment

Treatment of Tuberculosis [13]	ATS CDC IDSA	U.S.	2003	Adults Children	Treatment

Diagnostic Standards and Classification of Tuberculosis in Adults and Children [14]	ATS CDC IDSA	U.S.	2000	Adults Children	Diagnosis

Updated Guidelines for the Use of Nucleic Acid Amplification Tests in the Diagnosis of Tuberculosis [15]	CDC	U.S.	2009	Adults Children	Diagnosis

Updated Guidelines for Using Interferon Gamma Release Assays to Detect *Mycobacterium tuberculosis* Infection [16]	CDC	U.S.	2010	Adults Children	Diagnosis

Recommendations for Use of an Isoniazid-Rifapentine Regimen with Direct Observation to Treat Latent *Mycobacterium tuberculosis* Infection [17]	CDC	U.S.	2011	Adults Children	Treatment

European Union Standards for Tuberculosis Care [18]	ECDC ERS	Europe	2012	Adults Children	Diagnosis Treatment

Use of interferon-gamma release assays in support of TB diagnosis [19]	ECDC	Europe	2011	Adults Children	Diagnosis

Management of contacts of MDR TB and XDR TB patients [20]	ECDC	Europe	2012	Adults Children	Diagnosis Treatment

LTBI: latent tuberculosis infection or lasting immune responses to M. tuberculosis? A TBNET consensus statement [21]	TBNET	Europe	2009	Adults Children	Diagnosis

Clinical diagnosis and management of tuberculosis, and measures for its prevention and control [22]	NICE	U.K.	2011	Adults Children	Diagnosis Treatment

Diagnosis of tuberculosis in pediatrics. Consensus document of the Spanish Society of Pediatric Infectology (SEIP) and the Spanish Society of Pediatric Pneumology (SENP) [23]	SEIP SENP	Spain	2010	Children	Diagnosis

Consensus document for the treatment of pulmonar tuberculosis in children [24]	SEIP	Spain	2007	Children	Treatment

Recommendations of the Spanish Society for Pediatric Infectious Diseases (SEIP) on the management of drug-resistant tuberculosis [25]	SEIP	Spain	2009	Children	Treatment

Consensus document on treatment of tuberculosis exposure and latent tuberculosis infection in children [26]	SEIP	Spain	2006	Children	Treatment

Recommendations for Therapy, Chemoprevention and Chemoprophylaxis of Tuberculosis in Adults and Children [27]	DZK DGP	Germany	2012	Adult Children	Treatment

Childhood tuberculosis guidelines of the Southern African Society for Paediatric Infectious Diseases [28]	SASPID	South Africa	2009	Children	Diagnosis Treatment

Consensus Statement on Childhood Tuberculosis [29]	IAP	India	2010	Children	Diagnosis Treatment

Management of tuberculosis in children [30]	ASID APRG	Australasia	2000	Children	Treatment

Canadian Tuberculosis Standards 7^th^ Edition [31]	PHAC CTS CLA	Canada	2013	Adult Children	Diagnosis Treatment

Note. WHO: World Health Organization; IUATLD: International Union Against Tuberculosis and Lung Disease; AAP: American Academy of Pediatrics; ATS: American Thoracic Society; CDC: Center for Control Disease and Prevention; IDSA: Infectious Diseases Society of America; ECDC: European Center for Disease Control and Prevention; ERS: European Respiratory Society; NICE: National Institute for Health and Care Excellence; SEIP: Spanish Society for Paediatric Infectious Diseases; SENP: Spanish Society for Paediatric Respiratory Disease; DZK: German Central Committee against Tuberculosis; DGP: German Respiratory Society; SASPID: Southern African Society for Paediatric Infectious Diseases; IAP: Indian Academy of Pediatrics; ASID: Australasian Society for Infectious Diseases (Subgroup of Paediatric Infectious Disease); APRG: Australasian Paediatric Respiratory Group; PHAC: Public Health Agency of Canada; CTS: Canadian Thoracic Society; CLA: Canadian Lung Association.

### Diagnostic tests for *M. tuberculosis* infection

The demonstration of *M. tuberculosis* infection is a relevant part of the diagnosis of both TB disease and latent TB infection (LTBI). LTBI is defined as *M. tuberculosis* complex infection without clinical features or radiographic findings of TB disease [1,12,21,29]. Children with LTBI are at increased risk of developing active TB and becoming infectious. Therefore the identification of children latently infected with *M. tuberculosis* represents an important issue of TB preventive strategies.

Tuberculin skin test (TST) and interferon-gamma release assays (IGRAs) are immune-mediated methods currently available for identifying *M. tuberculosis* infection [1,6,9-12,14,16,19,21-23,28,29,31]. However, TST and IGRA are not able to distinguish between latent TB infection and active disease, and a negative result does not exclude the possibility of *M. tuberculosis* infection . Therefore, according to the AAP [12], both TST and IGRAs should not be considered a gold standard for the diagnosis of LTBI.

The TST is based on the evidence that *M. tuberculosis* infection promotes a delayed-type hypersensitivity reaction to antigenic components isolated from tubercle bacilli culture by protein precipitation (PPD), also known as tuberculin. All guidelines recommend that TST should be performed only in children who are at increased risk of *M. tuberculosis* infection (i.e. contact with people with contagious TB, HIV-infected children) or children with suspected TB disease. The preferred method of PPD administration is represented by Mantoux technique, which consists of an intradermal injection of 5 tuberculin units of PPD (0.1 ml) into the volar surface of the forearm, using a 27-gauge needle. The test should be read between 48 and 72 hours after the injection, measuring the transverse diameter of the induration. However, the interpretation of TST results is still controversial. The majority of the guidelines [1,9-11,23,28,31] affirms that TST should be considered positive if the diameter of the induration is > 10 mm in any child and > 5 mm in high risk children, including HIV-infected children and severely malnourished children. Otherwise the American guidelines [12,14] recommend three different cut-points. Five millimetres should be considered positive in high-risk children, including close contact with contagious people with TB, suspect of TB disease (i.e. chest radiograph suggestive of active or previous TB) and immunodeficiency (i.e. HIV-infected children, children receiving immunosuppressive therapy). In children at increased risk of disseminated TB disease (i.e. children younger than 4 years, children with other medical conditions) and children with likelihood of being infected with *M. tuberculosis* (i.e. children born in high prevalence regions or children who travel to high prevalence regions) the cut-off proposed is 10 mm. Eventually, TST > 15 mm is positive in children aged 4 years or older without any risk factors. All the guidelines agree that the TST result should be interpreted as positive regardless of Bacille Calmette-Guérin (BCG) vaccination, because there is no way of distinguishing between a positive TST due to *M. tuberculosis* infection and that caused by BCG vaccination.

Over the last decade new diagnostic tests have been developed for the detection of TB infection measuring, ex-vivo, the interferon-gamma released by T-cells in response to mycobacterial antigens (IGRAs) [32]. QuantiFERON-TB Gold-In tube (Cellestis, Victoria, Australia) and T-SPOT.TB (Immunotec, Oxford, United Kingdom) have been approved by the United States Food and Drug Administration [19] and licensed for commercial distribution in Europe [16] and several other countries [28,29,31].

All the recent guidelines are in agreement that IGRAs represent a relevant diagnostic tools for the identification of *M. tuberculosis* infection, except for one [9] which do not discuss the role of IGRAs in the diagnosis of TB infection. The CDC, the ECDC and the WHO have published specific recommendations focusing on the use of IGRAs in TB and LTBI diagnosis [6,16,19]. They analyze the sensitivity and specificity of the test, the costs and the role of IGRAs in the diagnosis of TB infection both in adults and children. Whereas the guidelines developed in high-income settings discuss extensively the use of IGRAs [21-23,31], those from middle or low-income countries only mention IGRAs [28,29].

All guidelines underline that few data concerning the use of IGRAs in childhood are available, but the IGRAs seem to perform well in children aged 5 years or older. The sensitivity of IGRAs for detecting TB infection in childhood is generally similar to TST, according also to recent meta-analysis [32-34]. The IGRAs specificity seems to be higher than TST, because the antigens used are absent in most pathogenic non-tuberculous mycobacteria, as well as in BCG strains. This characteristic explains the IGRAs advantage over TST in identifying a natural *M. tuberculosis* infection in settings with high non-tuberculous mycobacteria exposure and high BCG vaccination coverage [21,35]. Moreover boosting does not occur when the test is repeated, being particularly useful in BCG-vaccinated children. Anyway the IGRA tests have a reduced accuracy for the detection of *M. tuberculosis* infection in high-burden TB settings compared with low-burden TB settings [33,35]. Indeed IGRAs are unsuitable for resource-limited settings, being expensive and requiring sophisticated laboratory support and trained personal for accurate performance. According to that, the WHO [6] strongly recommends that IGRAs should not replace the TST for the detection of TB infection in children in low or middle-income countries, regardless of HIV status. On the contrary, the NICE guidelines [22] encourage the use of IGRAs and suggest considering those for all children aged 5 years or older whose TST shows positive results. Differently, European [19], Spanish [23], Canadian [31] and American [12,16] guidelines affirm that the selection of the most suitable test or combination of tests should be based on clinical data, as BCG status, history of contact with active TB or other risk factors for infection or progression of the disease (table 2).

**Table 2:** Recommendations for the use of Tuberculin Skill Test (TST) and Interferon-gamma Release Assay (IGRA) in children

	TST alone	IGRA alone	both TST and IGRA
**WHO **[6]	• Any child, irrespective of HIV status, in low and middle-income countries		

**NICE **[22]	• Children younger than 5 years	• BCG vaccinated children (> 5 years of age) • Children > 5 years of age, in an outbreak situation	• Children > 5 years of age whose TST is positive

**American **[12,16]	• Children younger than 5 years • Before initiation of immunosuppressive therapy	• BCG vaccinated children (> 5 years of age) • Children > 5 years of age who are unlikely to return for TST reading • Before starting immunosuppressive therapy	• The initial and repeat IGRA are indeterminate • The initial test (TST or IGRA) is negative and: - clinical suspicion for TB disease is moderate to high - risk of progression and poor outcome is high • The initial TST is positive and: - Children > 5 years of age who have received BCG vaccine - Non-tuberculous mycobacterial disease is suspected - Additional evidence are needed to increase compliance

**European **[19]	• Children younger than 5 years • Children > 5 years who are: - not HIV-infected - not BCG vaccinated	• BCG vaccinated children (> 5 years of age)	• HIV-infected children • Before starting immunosuppressive therapy (anti-TNFalfa inhibitor therapy)

**Spanish **[23]	• Any child		• The initial TST is negative and: - Immunocompromised children - High risk of infection, of progression to disease and of a poor outcome • The initial TST is positive and: - BCG vaccinated children - Risk factors are negative

**Canadian **[31]	• Whenever it is planned to repeat the test later to assess risk of new infection (i.e. conversions)	• People who have received BCG vaccination • People from groups that historically have poor rates of return for TST reading.	• High risk of infection, of progression to disease and of a poor outcome • The initial and repeat IGRA are indeterminate

Note. WHO: World Health Organization; BCG: Bacille Calmette-Guèrin

### Diagnostic tools for TB disease

All guidelines [1,9-12,14,18,22,23,28,29,31] agree that the isolation of *M. tuberculosis* complex from different specimens as sputum (natural or induced), gastric aspirates, bronchial washings, pleural fluid, cerebrospinal fluid, urine or biopsy tissue is the gold standard for the diagnosis of TB. Older children and adolescents frequently can produce sputum spontaneously or by induction with aerosolized hypertonic saline. Otherwise early-morning gastric aspirate, collected on three separate days, represents an option for young children and any child in whom sputum cannot be obtained. All guidelines underline that a microbiological confirmation should always be sought using all the resources available, although is well known that children are generally paucibacillary.

The first bacteriological evidence of the presence of mycobacteria in a clinical specimen is the detection of acid fast bacilli (AFB) in stained smears examined microscopically. This test gives an estimation of the numbers of bacilli excreted and therefore of the patient’s infectiousness. However, the results of AFB smears of gastric aspirates are often negative and also the presence of non-tubercolous mycobacteria, can cause false-positive results [12,31]. For these reasons all clinical specimens should be inoculated into culture media. Despite culture is the gold standard for laboratory confirmation of TB disease, *M. tuberculosis* is isolated from less than 50% of children and 75% of infants with pulmonary tuberculosis [12,31,36].

In low-income settings, where the facilities and resources are limited, bacteriological confirmation should be prioritized in children who have HIV infection, complicated or severe disease or suspected drug-resistant TB [1,28]. Moreover, the growth of the mycobacteria in culture is required not only for confirming the diagnosis and determining the mycobacteria species, but also for genotyping and drug susceptibility testing, according to all guidelines. Although all guidelines underline the importance of genotyping and drug susceptibility testing, only American and Spanish guidelines describe the methods used [14,23].

A relevant improvement in the direct identification of *M. tuberculosis* resulted from the introduction of nucleic acid amplification test (NAAT). The CDC [15] and the WHO [7] have recently published an updated guidelines for the use of NAAT in the diagnosis of TB in both children and adults, although some guidelines only explain briefly their potential implications [12,18,22,23,28,29,31], and other do not consider this topic [9-11]. The NAATs are rapid and accurate methods. They can provide the results within 24-48 hours, one or more weeks earlier than culture. Global sensitivity is greater than 95% in AFB-microscopy positive respiratory specimens and ranges between 50-80% in AFB-smear negative specimens [15]. Currently several assays are available, including line probe assays (LPAs) and NAATs using polymerase chain reaction (PCR). Real-time PCR is becoming increasingly available and several guidelines purpose its use for *M. tuberculosis* detection in respiratory specimens [7,12-15,18,22,23,28,31]. The CDC [15] recommends the use of NAAT in any patient with signs and symptoms of pulmonary TB, for whom a diagnosis has not yet been established or for whom the test result would change the case management. The NICE guideline [22], on the contrary, limits NAAT use only to sputum smear positive persons for whom the rapid confirmation of TB diagnosis would change their care. Other guidelines [23,28,31] underline the useful of NAAT, but they do not provide specific indications. However, all guidelines agree that NAATs do not replace AFB smear and culture, which should be always performed. Moreover the NAATs should not be used for treatment monitoring as they persist positive for long periods after therapy, being unable to distinguish live from dead bacilli.

However, evidence concerning NAATs accuracy for non-respiratory specimens are limited. Further research is needed before NAATs can be recommended for the diagnosis of TB in children who cannot produce sputum [12,15].

The NAATs are also able to detect drug resistance. If the AFB-smear is negative, as it frequently occurs in children, the NAAT of choice is the Xpert-MTB/RIF (Cepheid, Sunnyvale, California, USA) which is a real-time PCR assay detecting the 81-bp-core region of the RNA-polymerase b-subunit gene. This region, which is closed to M. tuberculosis specific DNA sequences, accounts for more than 95% of rifampicin (RIF) resistance. Therefore the Xpert-MTB/RIF can be used for the detection of RIF-resistance [37,38]. Additionally, given that rifampicin resistance is usually accompanied by isoniazid resistance, it can be used also as a rapid marker of multi-drug resistant (MDR) TB. According to these findings, the WHO [7,8] recommends the Xpert-MTB/RIF as the initial diagnostic test in children and adolescents suspected of having multidrug-resistant (MDR)-TB or HIV-associated TB. The WHO also suggests to consider it as a follow-on test to microscopy in settings where MDR-TB or HIV are of lesser concern, especially in further testing of smear-negative specimens. The ECDC guidelines [18] confirm the WHO recommendations.

Among radiological tools, all guidelines [1,9-12,18,22,23,28,29,31] are in agreement that chest radiography is an important part of the diagnostic workup of TB and should be performed in any child with suspected TB. Chest radiographic features vary widely, showing normal findings as well as lymphadenopathy, atelectasis, cavitary lesions, miliary disease and pleural effusion. There are no pathognomonic radiological sings of TB, but some radiological lesions strongly suggest TB, as miliary disease, hilar and paratracheal lymphadenopathy or cavitary lesions.

A general consensus exists also concerning the use of chest CT only for complicated cases, considering the high level of radiation exposure and the high costs related to its use.

The CT, as well as magnetic resonance, may also be very helpful in the evaluation of TB meningitis, osteoarticular disease, intra or extra-thoracic lymphadenopathy and pericardial TB.

### Treatment for TB disease

The major goals of anti-TB treatment are to cure the patient by eliminating most of the bacilli, prevent the development of drug resistance by using a drug combination regimen, prevent TB relapse by eliminating the quiescent bacilli and, finally, decrease the TB transmission to others.

The poor adherence to the anti-TB therapy represents the main contributory factor to treatment failure and emergence of TB drug-resistance. In order to improve the compliance to the treatment, all guidelines [1,5,9,12,13,18,22,24,25,27-31] emphasize the widespread use of the directly observed therapy (DOT), a patient-centred strategy in which patients are observed to ingest each dose of anti-TB drugs.

All guidelines are also in agreement that TB treatment should include an initial intensive phase, when three to four drugs are used in order to kill the majority of bacilli and prevent the emergence of drug resistance, and a continuation phase, when fewer drugs are given in order to eradicate quiescent bacilli. The first-line anti-TB drugs, their dosages and adverse events are summarized in table 3. The guidelines considered in the present review recommend the same standard treatment regimen for pulmonary TB (table 4). Children living in settings were HIV prevalence is high or isoniazid (INH)-resistance is high, or both, should be treated with a four drugs regimen (INH, RIF, pyrazinamide-PZA and ethambutol-EMB) for 2 months, followed by a two drugs regimen (INH, RIF) for 4 months. However, if the risk of drug-resistance and the HIV prevalence are low, EMB can be omitted, considering that it can cause retrobulbar neuritis and could be difficultly monitoring in children. Despite two recent systematic reviews [39,40] show that an intermittent short course of therapy is less likely to cure tuberculosis in childhood compare to daily therapy, several guidelines [1,5,9,12,13,22,24,27,30,31] purpose both daily and intermittent (twice or thrice weekly) dosing regimens, and major discrepancies are evidenced. The AAP [12], the ATS along with the CDC [13], as well as the APRG along with ASID [30] recommend a daily or twice-weekly regimen in both intensive and continuation phases. The NICE [22], otherwise, excludes the use of twice-weekly regimen for the treatment of active TB, suggesting a thrice-weekly intermittent regimens only for patients receiving DOT. The WHO [1], the IUATLD [9] and the CTS [31] limit a thrice-weekly regimen to children known to be HIV uninfected and living in settings with well established DOT, and they allow an intermittent regimen only during the continuation phase. Finally Spanish guidelines [24] agree that children who are known to be reliable with DOT may be considered for both twice and thrice-weekly regimens.

**Table 3:** First-line drugs used for treatment of tuberculosis in childhood [1,9,12,13,24,27-31]

	Daily dosage (maximum dosage)	Twice-thrice weekly dosage (maximum dosage)	Adverse Reactions
**Isoniazid (INH)**	10-15 mg/kg (300 mg)	20-30 mg/kg (900 mg)	Hepatotoxic effects , such as mild hepatic enzyme elevation and hepatitis, gastritis, peripheral neuropathy, hypersensitivity

**Rifampicin (RIF)**	10-20 mg/kg (600 mg)	10-20 mg/kg (600 mg)	Orange discoloration of secretions and urine, vomiting, hepatitis, influenza-like reaction, thrombocytopenia

**Pyrazinemide (PZA)**	30-40 mg/kg (2 g)	50 mg/kg (2 g)	Hepatotoxic effects, hyperuricemia, arthralgia, gastrointestinal tract disturbances

**Ethambutol (EMB)**	15-25 mg/kg (2,5 g)	50 mg/kg (2,5 g)	Optic neuritis with decreased red-green colour discrimination and visual acuity, gastrointestinal disturbances, hypersensitivity

**Table 4:** Recommendations for treatment of TB in childhood [1,8,9,12,13,17,18,21,22,24,27-31]

	Intensive phase (duration)	Continuation phase (duration)
**TB disease (except meningitis and osteoarticular TB) in HIV-uninfected children with low risk of INH-resistance**	INH + RIF + PZA (2 months)	INH + RIF (4 months)

**TB disease (except meningitis and osteoarticular TB) in HIV-infected children and/or children with high risk of INH-resistance**	INH + RIF + PZA + EMB (2 months)	INH + RIF (4 months)

**Meningitis and osteoarticular TB**	INH + RIF + PZA + EMB (2 months)	INH + RIF (10 months)

**INH-monoresistance TB**	RIF + PZA + EMB (2 months)^§^	RIF + PZA + EMB (4-7 months)^§^
	
	RIF + PZA + EMB (2 months)^#^	RIF + EMB (10 months) ^#^
	
	RIF + PZA + EMB + FQN (2 months) ^#^	RIF + EMB + FQN (4-7 months) ^#^

**RIF-monoresistance TB**	INH+ PZA + EMB + FQN (2 months) ^§^	INH + EMB + FQN (10-16 months) ^§^
	
	INH + PZA + EMB (2 months) ^#^	INH + EMB (16 months) ^#^

**MDR-resistance TB**	Treatment regimens should be based on the drug susceptibility pattern of the *M. tuberculosis* isolated from child specimens or, more frequently, from the source case specimens.	

**LTBI**	INH (6-9 months) ^§^	
	
	INH + RIF (3 months) ^#^	
	
	INH + RPT (weekly for 12 weeks) ^#^	

**INH-monoresistance LTBI**	RIF (4-6 months)	

Note: § recommended regimen; # alternative regimen; TB: Tuberculosis; INH: Isoniazid; RIF: Rifampicin; PZA: Pyrazinamide; EMB: Ethambutol; FQN: Fluoroquinolones; MDR: Multi- Drug Resistance; RPT: Rifabutin.

A general consensus exists concerning treatment regimes for extra-pulmonary TB (table 4): except for meningitis and osteoarticular TB, the standard recommended regimen (INH + RIF + PZA + EMB for 2 months, followed by INH + RIF for 4 months) should be started. Otherwise, the guidelines strongly recommend 12 months of therapy for meningeal TB [1,5,9,12,13,22,27,30,31] and osteoarticular TB [1,5,9,22,27,30,31].

Among drug-resistance TB, only few guidelines [1,8,12,25,30,31] provide specific recommendations, probably due to the lack of evidence concerning this issue in childhood. Recommended regimens [8,12,25,30,31] for INH-monoresistance and RIF-monoresistance TB are listed in table 4. Despite the evidence concerning safety and efficacy in TB treatment of fluoroquinolones in children are few, all guidelines recommend their introduction in the treatment of monodrug-resistance TB, considering the indirect evidence from cystic fibrosis and osteomyelitis treatment in children.

The AAP [12] define the MDR as a TB infection or disease caused by a strain of *M. tuberculosis* complex that is resistant to at least INH and RIF. For MDR-TB cases, all guidelines [1,8,18,12,25,30,31] are in agreement that the treatment should be based on the drug susceptibility pattern of the *M. tuberculosis* isolated from child specimens or, more frequently, from the source case specimens. All guidelines also affirm that treatment regimen should include at least four anti-TB drugs to which the organism isolated is susceptible [1,8,18,12,25,31], except for Australasian one that recommends the use of at least two drugs certain to be effective [30]. Additionally treatment duration should be prolonged and ranges from 12 to 24 months in the different guidelines [1,8,18,12,25,30,31]. Second-line drugs used in paediatric TB are listed in table 5.

**Table 5:** Second-line drugs used for TB treatment in children [8,12,13,25,27,30,31]

	Daily dosage (maximum dosage)	Adverse Reactions
**Streptomycin**	20-40 mg/kg (1 g)	Auditory, vestibular and renal toxicity, rash

**Amikacin** **Kanamycin** **Capreomycin**	15-30 mg/kg (1 g)	Auditory, vestibular and renal toxicity

**Ethionamide**	15-20 mg/kg/die, given in 2-3 divided doses (1 g)	Hepatotoxic effects, gastrointestinal tract disturbances, neurotoxicity, hypersensitivity and hypothyroid

**Para-amino salicylic acid**	200-300 mg/kg/die, given in 2-4 divided doses (10 g)	Hepatotoxic effects, gastrointestinal tract disturbances, hypersensitivity

**Cycloserine**	10-20 mg/kg, given in 2-divided doses (1 g)	Psychosis, personality changes, seizure, rash

**Moxifloxacin**	10 mg/kg (400 mg)	Theoretical effect on growing cartilage, gastrointestinal tract disturbances, rash, headache, restlessness
	
**Levofloxacin**	10 mg/kg (1 g)	

**Linezolid**	10 mg/kg (1,2 g)	gastrointestinal tract disturbances, peripheral neuropathy, thrombocytopenia

### Treatment for LTBI

After exposure to an active case, children are more likely to develop tuberculosis than adults, hence contact screening and chemoprophylaxis are particularly important. Several guidelines discuss LTBI and its treatment [1,12,13,17,18,21,26-31] and all of those are in agreement that any child who has an history of TB exposure, positivity of TST and/or IGRA, but not evidence of active tuberculosis, should start the treatment for LTBI (table 4).

Isoniazid represents the preferred drug for children with LTBI, unless INH-resistance is suspected, and the efficacy approaches about 100% if the adherence to therapy is adequate [12]. All guidelines [1,12,13,17,18,21,26-31] suggest using isoniazid alone for LTBI treatment in childhood although minor differences concern the treatment duration, which ranges from 6 months [1,22,26,28-30] to 9 months [12,13,17,21,27,31]. Additionally the NICE [22] and the Southern African [28] guidelines purpose 3 months of INH and RIF as alternative therapy regimen. Recently the CDC has published recommendations for the use of a new combination regimen of rifapentin (RPT) and INH to be administered weekly for 12 weeks as DOT [17]. Although the evidence on tolerability and efficacy of this combination in childhood is lacking, it can be considered in selected cases, if the compliance to treatment is low and the hazard of TB is high.

Rifampicin alone for 4-6 months is recommended for the treatment of LTBI if a resistance to INH is strongly suspected or demonstrated [1,12,13,21,26,27,30,31]. However, on the basis of the currently available evidences, second-line drugs are not recommended for the chemoprophylaxis in MDR-TB contacts.

## Conclusions

The present systematic review has identified 27 national and international guidelines concerning the management of paediatric tuberculosis [1,5-31].

There are several discrepancies on the diagnosis workup of TB. Major differences concern the TST interpretation. Most of the guidelines [1,9-11,23,28,31] establish a cut-point of 10 mm for any child and 5 mm for high risk children, although the American guidelines [12,14] increase the cut-point to 15 mm for children without any risk factors and to 10 mm for children with minor risk factors, maintaining the diameter of 5 mm only for high risk population. Considering that TST sensitivity and specificity are influenced by the cut-off used [21], differences among guidelines are not merely academic but have important consequences for clinical practice. Recommendations also differ substantially regarding the use of TST and IGRA for the diagnosis of TB infection. The guidelines from high-income countries [12,16,22,23,19,31] widely promotes the IGRA use, whereas the WHO [6] strongly recommends that IGRAs should not replace the TST for the detection of TB infection in children in low or middle-income countries. These findings could be explained considering that IGRA has a reduced accuracy in high-burden TB settings compared with low-burden TB settings [33,35], is expensive and requires sophisticated laboratory support and trained personal [6,16,21].

A general consensus exists, on the contrary, on the microbiological confirmation of TB diagnosis, despite in low-income settings the mycobacterial culture is not performed routinely and is limited to HIV-infected children or severe disease. Although all guidelines underline the importance of susceptibility drug testing and genotyping, the guidelines from low-income countries do not strongly recommend these tests routinely to all patients, probably because of costs and lack of facilities. Finally the use of NAATs for the direct detection of *M. tuberculosis* is still controversial. Whereas the CDC [15] and the WHO [7] have recently published an updated guidelines focusing on the use of NAATs, the majority of guidelines [12,18,23,28,29,31] do not provide specific recommendations on their use. Moreover, evidence concerning NAATs accuracy for non-respiratory specimens are limited and further research is needed before NAATs can be recommended for the diagnosis of TB in children who cannot produce sputum [12,15].

Despite advances in TB diagnostic tool have been reached over the last decade, a lack of uniformity in their availability, indication and interpretation has relevant consequences for clinical practice. Further research is needed to identify a reliable and reproducible diagnostic workup not only to improve individual case management, but also to provide a valid basis for epidemiological data analysis, drug efficacy evaluation and improving clinical trials.

The recommendations on TB treatment are otherwise homogeneous and only minor discrepancies are evidenced in the present review. The main point of disagreement is represented by the daily and intermittent (twice or thrice weekly) regimens recommended, which vary widely between different guidelines. The lack of evidence among the drugs metabolism and the efficacy of intermittent treatment regimes in childhood is probably the main reason of this finding. Further studies need to be performed in order to clarify this issue. Finally, the poor adherence to the therapy, which is often too long, represents a relevant concern, especially in low-income settings where DOT is not always guarantied. The combination regimen of RPT and INH, recently proposed by the CDC for LTBI, could be a valid alternative in these patients. Data on efficacy and safety of this combination are needed before address new recommendations. Moreover, new treatment regimens, indicated also for active TB, should be analyzed in future studies in order to improve the treatment compliance.

## List of abbreviations used

AAP: American Academy of Pediatrics; AFB: Acid Fast Bacilli; APRG: Australasian Paediatric Respiratory Group; ASID: Australasian Society for Infectious Diseases; ATS: American Thoracic Society; BCG: Bacille Calmette-Guérin; CDC: Center for Disease Control and Prevention; CLA: Canadian Lung Association; CTS: Canadian Thoracic Society; DGP: German Respiratory Society; DOT: Directly Observed Therapy; DZK: German Central Committee against Tuberculosis; ECDC: European Center for Disease Control and Prevention; EMB: Ethambutol ; ERS: European Respiratory Society; FQN: Fluoroquinolones; IAP: Indian Academy of Pediatrics; IDSA: Infectious Diseases Society of America; IGRA: Interferon-gamma Release Assays; IHN: Isoniazid; IUATLD: International Union Against Tuberculosis and Lung Disease; LPAs: Line Probe Assays; LTBI: Latent Tuberculosis Infection; MDR: Multi Drug Resistance; NAAT: Nucleic Acid Amplification Test; NICE: National Institute for Health and Care Excellence; PCR: Polymerase Chain Reaction; PHAC: Public Health Agency of Canada; PPD: Purified Protein Derivate, Tuberculin; PZA: Pyrazinamide; RIF: Rifampicin; RPT: Rifabutin; SASPID: Southern African Society for Paediatric Infectious Diseases; SEIP: Spanish Society for Paediatric Infectious Diseases; SENP: Spanish Society for Paediatric Respiratory Disease; TB: Tuberculosis; TST: Tuberculin Skin Test; WHO: World Health Organization

## Competing interests

The authors declare that they have no competing interests.

## Funding

Italian Health Ministry /Young Research Project has provided the funding for publication of the present systematic review.

## Declarations

This article has been published as part of *BMC Infectious Diseases* Volume 14 Supplement 1, 2014: Highlights in Pediatric Tuberculosis. The full contents of the supplement are available online at http://www.biomedcentral.com/bmcinfectdis/supplements/14/S1
